# P41 Major bacteria species surface contaminants in hospitals of Littoral Region, Cameroon

**DOI:** 10.1093/jacamr/dlac004.040

**Published:** 2022-02-16

**Authors:** Wandji Jonas Merlin Takemegni, Nguedia Jules Clement Assob, Jeurome Ateudjeu, Orock George Enow, Ngowe Marcelin Ngowe

**Affiliations:** Department of Biomedical Sciences, Faculty of Health Sciences, University of Buea, Cameroon; Department of Medical Laboratory Sciences, New-Bell District hospital, Douala, Cameroon; Department of Biomedical Sciences, Faculty of Health Sciences, University of Buea, Cameroon; M. A. SANTE (Meilleur Accès aux Soins de Santé/Better Access to Health Care), Yaoundé, Cameroon; Department of Biomedical Sciences, University of Dschang, Dschang, Cameroon; Division of Health Operations Research, Ministry of Public Health, Yaoundé, Cameroon; Department of Biomedical Sciences, Faculty of Health Sciences, University of Buea, Cameroon; Department of Biomedical Sciences, Faculty of Health Sciences, University of Buea, Cameroon; Department of Clinical Sciences, Faculty of Medicine and pharmaceutical Sciences, University of Douala, Cameroon

## Abstract

**Background:**

Globally, the levels of healthcare-associated infections (or nosocomial infections) are high and especially those due to bacteria are significant and costly. Healthcare environments provide a worrying reservoir for spreading infections. According to WHO, low and middle-income countries may be particularly at risk. Hence, the need to perform timely assessment of surface contamination of bacterial origin, in four different hospitals in major units of the Littoral Region, Cameroon.

**Methods:**

A cross-sectional and descriptive study was conducted from December 2018 to May 2019. A simple random sampling was used to swab 10 selected equipment items (treatment tables, operating tables, delivery tables, office tables, anaesthesia equipment, surgical aspirators, oxygen concentrators, wheelchairs, patients and office chairs) and 10 materials (fans, patient bedside tables, patient bed rails, trolleys, door handles, negatoscopes, baby scales, air conditioners, antiseptic container boxes and antiseptic container covers). In the mornings after disinfection but before start of work in each unit, we collected all selected equipment and materials using sterile swabs moistened in sterile normal saline, which were immediately transported to the microbiology laboratory for processing. After inoculation in four agar media consecutively (three sector petri dish with different media: eosin methylene blue, CLED and mannitol salt agar and the blood agar media in one segmented petri dish) and incubated in appropriate conditions. Identification and confirmation were based on morphological characteristics of bacteria colonies, microscopy and biochemical methods using API staph, strepto and 20ETM gallery (bioMérieux).

**Results:**

Of a total of 236 samples collected, 119 (50.4%) showed bacterial growth, 33 different species of which 62/119 (52.10%) were Gram-positive cocci and 57/119 (47.90%) Gram-negative bacilli. *Staphylococcus aureus* 45/119 (37.81%), *Escherichia coli* 6/119 (5.04%) and *Acinetobacter* spp. 4/119 (3.36%) were the most common contaminants. Patient bedside tables, office chairs and patient bed rails were the highest contaminants respectively 14/119 (11.76%), 13/119 (10.92%) and 12/119 (10.08%). Emergency units were the main contaminated area for Gram-positive cocci, 12/62 (19.35%) majority *S. aureus* 9/62 (14.51%) as most as Gram-negative non-Enterobacteriaceae 11/31 (35, 48%) with predominance of *Chryseomonas meningoseptica* and *Mannheimia haemolytica* both 2/31 (6.45%). Therefore for Gram-negative bacilli Enterobacteriaceae family, the highest level of bacterial isolates was recovered in laboratory 7/26 (26.92%), in which *E. coli* was predominantly reported 4/26 (15.38%). However, *S. aureus* was the only species found in all hospitals and units.

**Conclusions:**

Hospital environment is a serious reservoir of bacteria. This work will help clinical care and decision making to take appropriate actions to improve sanitation and ensure control measures to limit the spread of hospital-acquired infections.
